# Method for assessing visual saliency in children with cerebral/cortical visual impairment using generative artificial intelligence

**DOI:** 10.3389/fnhum.2024.1506286

**Published:** 2025-01-17

**Authors:** Kate Matsunaga, Kleanthis Avramidis, Mark S. Borchert, Shrikanth Narayanan, Melinda Y. Chang

**Affiliations:** ^1^Keck School of Medicine, University of Southern California, Los Angeles, CA, United States; ^2^Viterbi School of Engineering, University of Southern California, Los Angeles, CA, United States; ^3^Division of Ophthalmology, Department of Surgery, Children’s Hospital Los Angeles, Los Angeles, CA, United States

**Keywords:** cortical visual impairment, cerebral visual impairment, eye tracking, generative artificial intelligence, functional vision assessment

## Abstract

Cerebral/cortical visual impairment (CVI) is a leading cause of pediatric visual impairment in the United States and other developed countries, and is increasingly diagnosed in developing nations due to improved care and survival of children who are born premature or have other risk factors for CVI. Despite this, there is currently no objective, standardized method to quantify the diverse visual impairments seen in children with CVI who are young and developmentally delayed. We propose a method that combines eye tracking and an image-based generative artificial intelligence (AI) model (SegCLIP) to assess higher- and lower-level visual characteristics in children with CVI. We will recruit 40 CVI participants (aged 12 months to 12 years) and 40 age-matched controls, who will watch a series of images on a monitor while eye gaze position is recorded using eye tracking. SegCLIP will be prompted to generate saliency maps for each of the images in the experimental protocol. The saliency maps (12 total) will highlight areas of interest that pertain to specific visual features, allowing for analysis of a range of individual visual characteristics. Eye tracking fixation maps will then be compared to the saliency maps to calculate fixation saliency values, which will be assigned based on the intensity of the pixel corresponding to the location of the fixation in the saliency map. Fixation saliency values will be compared between CVI and control participants. Fixation saliency values will also be correlated to corresponding scores on a functional vision assessment, the CVI Range-CR. We expect that fixation saliency values on visual characteristics that require higher-level processing will be significantly lower in CVI participants compared to controls, whereas fixation saliency values on lower-level visual characteristics will be similar or higher in CVI participants. Furthermore, we anticipate that fixation saliency values will be significantly correlated to scores on corresponding items on the CVI Range-CR. Together, these findings would suggest that AI-enabled saliency analysis using eye tracking can objectively quantify abnormalities of lower- and higher-order visual processing in children with CVI. This novel technique has the potential to guide individualized interventions and serve as an outcome measure in future clinical trials.

## 1 Introduction

Cerebral/cortical visual impairment (CVI) is a leading cause of pediatric visual impairment in developed countries, and is increasingly diagnosed in developing nations due to improved care and survival of children who are born premature or have other risk factors for CVI, such as hypoxic–ischemic encephalopathy (Chang and Borchert, 2020; Chang and Borchert, 2024; Kong et al., 2012; Chong et al., 2019; Pehere et al., 2019). Although the United States does not currently have a registry of children with visual impairment, a recent study from Denmark found that the prevalence of visual impairment was 1.6 per 1,000 children <18 years of age, with CVI accounting for 36% of children with visual impairment in 2022 (Kessel et al., 2024). In India, CVI was diagnosed in 44% of young children with profound visual impairment seen at a tertiary eye center (Pehere et al., 2019).

CVI encompasses a wide range of visual impairments due to structural and/or functional brain abnormalities that affect visual pathways in the developing brain (Chang et al., 2024). While children with CVI may have comorbid ocular conditions, the visual dysfunction is worse than expected for the degree of ocular pathology (Chang et al., 2024; Sakki et al., 2018). Causes of CVI include hypoxic–ischemic encephalopathy in children born at term, seizures with epileptic encephalopathy, prematurity with periventricular leukomalacia, hydrocephalus, meningoencephalitis, trauma, metabolic and genetic disorders, among others (Chang and Borchert, 2020; Chang et al., 2022; Huo et al., 1999). A wide variety of genetic disorders have been associated with CVI, including Down syndrome, Bosch Boonstra Schaaf optic atrophy syndrome, tuberous sclerosis, Angelman syndrome, and many others (Chang and Borchert, 2020). In the case of these neurogenetic syndromes and other causes of CVI, there may be overlap with other neurodevelopmental conditions such as autism spectrum disorder (ASD). However, the recent working definition of CVI from the National Institutes of Health (NIH) CVI Workshop specifies that “while CVI may be comorbid with other neurodevelopmental disorders, CVI is not primarily a disorder of language, learning, or social communication” (Merabet et al., 2017). Although children with ASD are known to have abnormal visual social attention which can be measured with eye tracking (Falck-Ytter et al., 2013), children with CVI have additional deficits of visual function and processing that cannot be attributed to ASD alone.

Due to involvement of visual pathways in the brain, children with CVI often exhibit visual characteristics that differ from those with purely ocular disorders (Chang and Borchert, 2020; Jan et al., 1987). Similar to children with ocular causes of visual impairment, children with CVI may have abnormalities of lower-order visual function (aspects of vision that are processed in the visual pathway from the eye to the primary visual cortex, such as visual acuity and contrast sensitivity) (Good, 2001; Good et al., 2012). However, children with CVI are unique in that they may have impairments of higher-order visual processing (localized to visual association areas in the brain) (Dutton et al., 1996). Examples include abnormalities of dorsal stream processing leading to dysfunction in visuospatial orientation and complex motion perception, as well as disorders of recognition such as prosopagnosia (difficulty with recognizing faces) (Chang and Borchert, 2020; Dutton et al., 1996, 2006; Ahmed and Dutton, 1996; Chandna et al., 2021a, 2021b; Bauer et al., 2023; Dutton, 2009; Ortibus et al., 2012). Children with CVI also have challenges with visual search and recognition of objects against complex backgrounds (Jan et al., 1987; Manley et al., 2022). While lower-order visual deficits may be elicited by standard pediatric ophthalmologic examination techniques, diagnosis of higher-order visual deficits often necessitates neuropsychological assessment, which generally requires children to have a developmental age of at least 3 years in order to have the cognitive ability to understand and respond to questions about visual perception.

**Figure 1:**
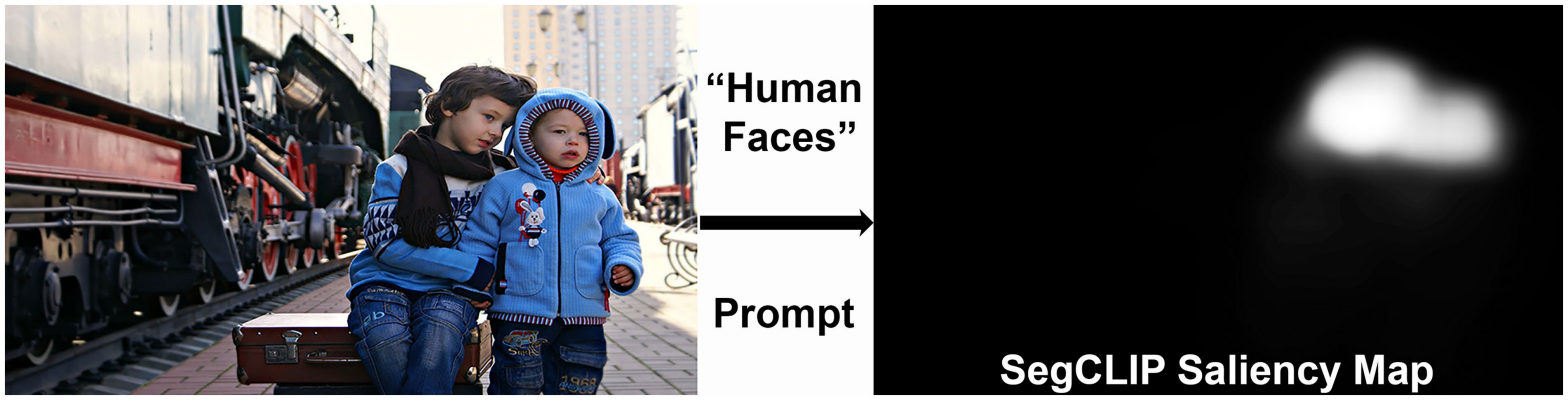
Example of a “human faces” saliency map generated by prompting the SegCLIP generative artificial intelligence (AI) model. The saliency map highlights the faces of the children in the image (Reproduced from Victoria Borodinova via https://www.publicdomainpictures.net/en/index.php, licensed under CC0).

Currently, there is no objective test that measures the diverse visual deficits that occur in CVI and is applicable to young, developmentally delayed children (Chang and Borchert, 2021). We propose a method to assess visual attention to lower- and higher-order visual characteristics in children with CVI by utilizing eye tracking technology combined with artificial intelligence (AI) for saliency analysis. Eye tracking has previously been used to assess visual acuity in young children with CVI, as well as visual search in older individuals with CVI (Chang and Borchert, 2024; Manley et al., 2022). Additionally, eye tracking has previously been used in children with CVI to assess visual orienting functions, especially reaction time to fixation when presented targets of interest such as cartoon faces and moving dots (Ben Itzhak et al., 2023). In the present study, we will assess eye tracking patterns using saliency analysis, a computer vision technique to determine which aspects of an image attract a viewer’s attention. Saliency analysis in older teenagers and young adults with CVI has demonstrated that visual search patterns are primarily driven by bottom-up, low-level visual features (including color, orientation, and intensity), rather than top-down, higher-level features that are dependent on semantic associations between words and objects (Walter et al., 2024). Because the previous study used saliency models that combine multiple features, there remains a question of which individual visual characteristics are specifically affected in CVI, and to what degree. For this study, we will use an image-based generative artificial intelligence (AI) model, SegCLIP (Luo et al., 2023) to generate saliency maps that highlight individual features of interest (Figure 1) (Avramidis et al., 2024). By comparing eye tracking patterns in children with CVI and age-matched controls to these saliency maps, we will quantify differences in visual attention to lower- and higher-order visual characteristics between the two groups.

The goals of this study are (1) to assess whether eye tracking combined with AI-generated saliency models can quantify differences in visual attention to lower- and higher-order visual characteristics in children with CVI and (2) to correlate eye tracking measures to corresponding scores on the CVI Range-CR functional vision assessment (Chang et al., 2022).

## 2 Methods

This study has been approved by the local institutional review board (IRB) and will adhere to the Declaration of Helsinki and the US Health Insurance Portability and Accountability Act of 1996. Informed consent will be obtained from the parent or legal guardian of all participants.

### 2.1 Participants

Children with CVI and age-matched controls between the ages of 12 months and 12 years will be recruited from our pediatric neuro-ophthalmology clinic and a web-based recruitment service (Buildclinical.com). The CVI group will include children who are developmentally delayed and unable to participate in standard neuropsychological assessments of visual perception. The minimum age is 12 months because some practitioners do not diagnose CVI until this age in order to confirm that the child does not have delayed visual maturation (Good et al., 1994). We will include children with a chronological age up to 12 years so that our sample is representative of the age range of children with CVI diagnosed in the community; challenges with measuring higher-order visual processing deficits may lead to CVI being diagnosed late or unrecognized (Williams et al., 2021). Diagnosis of CVI will be based on reduced visual function with a normal eye exam (with the exception of mild optic atrophy) in the context of neurologic pathology affecting structure or function of the optic radiations, primary visual cortex, and/or visual association areas. A neurologic diagnosis will be made in conjunction with a pediatric neurologist based on neurologic examination, neuroimaging, and/or genetic testing, as appropriate. We will allow patients with mild optic atrophy to enroll in the study due to the high proportion of children with CVI in our clinic with this ocular comorbidity (approximately 30%). However, we will perform subgroup analysis to determine whether the inclusion of these patients impacted the study results.

Exclusion criteria for the CVI group will include photosensitive epilepsy, any oculomotor abnormality that would preclude accurate assessment of afferent visual function based on direction of eye gaze (e.g., oculomotor apraxia, the presence of which will be assessed clinically), and binocular visual acuity worse than 3 cycles per degree (cpd) based on preferential looking testing during eye tracking. We chose this threshold for visual acuity because Fourier analysis of the visual stimuli in our experimental protocol revealed that greater than 99% of each image was represented by spatial frequencies above 3 cpd.

The control group will include children with no known neurologic, neurodevelopmental, or visual disorder, other than corrected refractive error. The presence of neurologic and neurodevelopmental disorders will be determined based on medical history, and children born prematurely will be excluded. Controls will be required to have a normal screening eye exam (including age-normal visual acuity) in order to participate.

### 2.2 Sample size calculation

In our preliminary studies, the saliency characteristic with the least difference between groups was orientation. Fixation saliency values were 48 in the CVI group and 43 in the control group, with a standard deviation of 8. Using these values, with a power of 80% and alpha of 0.05, the sample size required to demonstrate a significant difference between groups is 80 (40 per group). We will aim to recruit 100 participants to account for up to 20% attrition.

### 2.3 Materials and equipment

#### 2.3.1 Eye tracking

We will use an EyeLink^®^ 1,000 Plus eye tracker (SR Research, Ontario, Canada) to record the direction of eye gaze as X and Y coordinates of each eye at 500 Hz. The Eyelink software automatically identifies fixations and saccades based on the following parameters. Fixations are defined as periods when the gaze is stable within 0.1 degree for at least 100 ms. Saccades are detected when velocity exceeds 30 degrees/s and acceleration exceeds 8,000 degrees/s^2^ over an amplitude of greater than 0.1 degree.

During the eye tracking experiment, participants will view a series of still images including naturalistic and cartoon pictures of landscapes, animals, and people displayed on a 24-inch computer monitor. These images are interspersed with psychophysical stimuli to assess other visual characteristics, including visual acuity. Visual acuity will be measured using preferential looking during eye tracking, which we have previously found to be reliable and valid in children with CVI (Chang and Borchert, 2024).

#### 2.3.2 Functional vision assessment (CVI range-CR)

**Figure 2:**
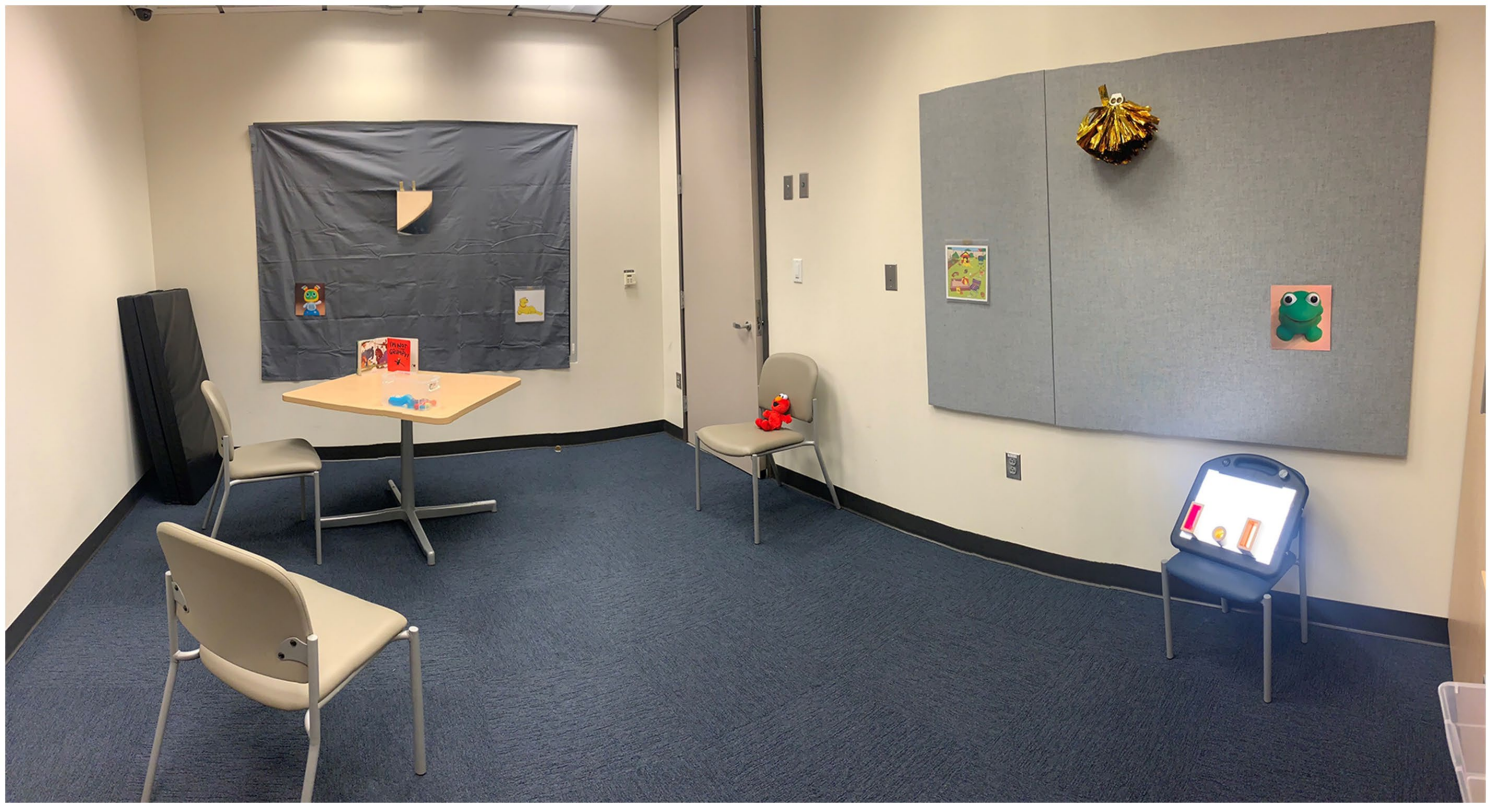
Standard room configuration for the CVI Range-CR functional vision assessment. Reproduced from Chang et al. (2022), with permission from BMJ Publishing Group Ltd.

A subset of participants with CVI will undergo functional vision assessment with the CVI Range-CR, a standardized version of the CVI Range developed for clinical research purposes and conducted by a trained neuropsychologist (Chang et al., 2022). Because the CVI Range-CR is only available in English, this assessment will only be performed in children from English-speaking families. Additionally, because the CVI Range-CR assessment is substantially longer than eye tracking (approximately 1 h), some participants who consent to eye tracking are expected to be unable or unwilling to complete the CVI Range-CR assessment. Based on our initial recruitment experience, we expect that approximately 50% of participants who complete eye tracking will be eligible and consent to undergo the CVI Range-CR. This assessment is conducted in a room with a standard configuration, including specific materials placed on the walls (Figure 2) and others used for interactions with the child (Table 1). The CVI Range-CR consists of an interview of parents/caregivers and direct assessment while a child is performing activities to elicit the ability to use vision in everyday life. Children are scored on 10 CVI characteristics, which results in two scores via the “Across-CVI Characteristics Assessment Method” (Score 1) and “Within-CVI Characteristics Assessment Method” (Score 2, Supplementary material). For the purposes of this study, we will use these overall scores as well as scores on individual items of the “Within-CVI Characteristics Assessment Method” (Supplementary material).

**Table 1:** Materials used for direct assessment in the CVI Range-CR.

Flashlight with red light
Brightly colored strings of beads of various colors (“Mardi gras” beads)
Translucent neon Slinky toys
Clear plastic colored blocks
A dozen plastic animals (approximately 1″ height)
Two stuffed animals (10″ height): red Elmo and yellow Big Bird
Musical toy (e.g., Fisher-Price BeatBo)
Black tray
Colorful patterned fabric
Picture book (see description in text)
Light box
CVI Complexity Sequence Cards (American Printing House, Louisville, KY)
iPad with apps (Tap-N-See Now, CVIHumanFace)
Handheld mirror (8.5×11″)
Dimmable floor lamp

Reproduced from Chang et al. (2022), with permission from BMJ Publishing Group Ltd.

### 2.4 Data acquisition

#### 2.4.1 Eye tracking

During eye tracking, participants will sit (alone, in a stroller, or in a parent’s lap) 60 cm from a computer monitor with the eye tracking camera attached at the bottom of the screen. The room lights will be off, and blackout curtains will be placed on the windows. Families will be instructed to avoid distracting the child by speaking, unless necessary to direct gaze to the screen. A target sticker will be placed at the center of the child’s brow to facilitate recording. Eye tracking will be performed binocularly, with participants wearing their habitual spectacles. In patients with strabismus and a consistent fixation preference, we will record from the fixating eye. However, if a child has strabismus with alternating fixation, we will patch one eye to prevent switching fixation. After three-point calibration, the eye tracking experiment will commence. The visual stimuli described above (Materials and Equipment section) will be shown on the computer monitor and the participant’s direction of eye gaze will be tracked for a total of 10 min. No instructions will be given to the child other than to continue to watch the screen and try to avoid head movement. During the eye tracking session, a warning sound will be played if the eye gaze is detected off the screen. Experimenters will attempt to redirect gaze to the screen verbally or using a toy if needed, and will reposition the head if necessary to regain tracking of the eyes.

#### 2.4.2 CVI range-CR

The CVI Range-CR functional vision assessment will be performed and scored by a neuropsychologist, as described above.

### 2.5 Data analysis

#### 2.5.1 Saliency maps

In order to assess eye gaze to lower- and higher-order visual characteristics, we will use AI to generate saliency maps of the stimulus images. Compared to other saliency maps that have been used in CVI (Walter et al., 2024), a unique feature of our study is that we will use generative AI to create maps that highlight a single feature of interest, rather than multiple features. Thus, we can assess the degree to which individual lower- and higher-level visual characteristics are impacted in children with CVI. SegCLIP, the generative AI model used in this study, consists of a text and image encoder (Dutton, 2009). It can take free language prompts and an input image to highlight specific regions within the image in the form of saliency maps (Figure 1) (Avramidis et al., 2024). The resulting grayscale maps are then smoothed with a Gaussian filter and normalized such that each pixel is assigned an intensity value from 0 to 255. SegCLIP can also perform differential saliency analysis wherein two prompts with opposite attributes can be inputted in order to enhance the accuracy and nuances of the saliency prediction. Each prompt will initially generate its own saliency map. The subtraction of the two maps will generate the final saliency map used for the differential analysis (Avramidis et al., 2024). Each saliency map will be manually checked for accuracy to ensure appropriate areas are highlighted.

The saliency maps that are currently planned include: color (red, yellow, green, and blue); visual field maps (central vs. peripheral screen, upper vs. lower screen, and right vs. left screen); luminance or intensity; contrast; orientational patterns (prompted as “bars” or “stripes”); background; depth; animals; human faces; human bodies; movement; and complexity.

Additionally, we will create saliency maps using DeepGazeII, a saliency model that predicts salient object fixations using a deep neural network pretrained on object recognition via the SALICON dataset, which includes 10,000 images annotated by humans (Kümmerer et al., 2016). The DeepGazeII model predicts the fixations of (presumably) typically developing adults with high accuracy (AUC 0.88). Thus, we will use DeepGazeII saliency maps as an indicator of typical adult visual attention.

#### 2.5.2 Comparison of CVI and control participants

Fixation maps of CVI and control participants will be combined with saliency maps to calculate fixation saliency values, as described below. Subsequently, fixation saliency values will be compared between CVI and control participants.

**Figure 3:**
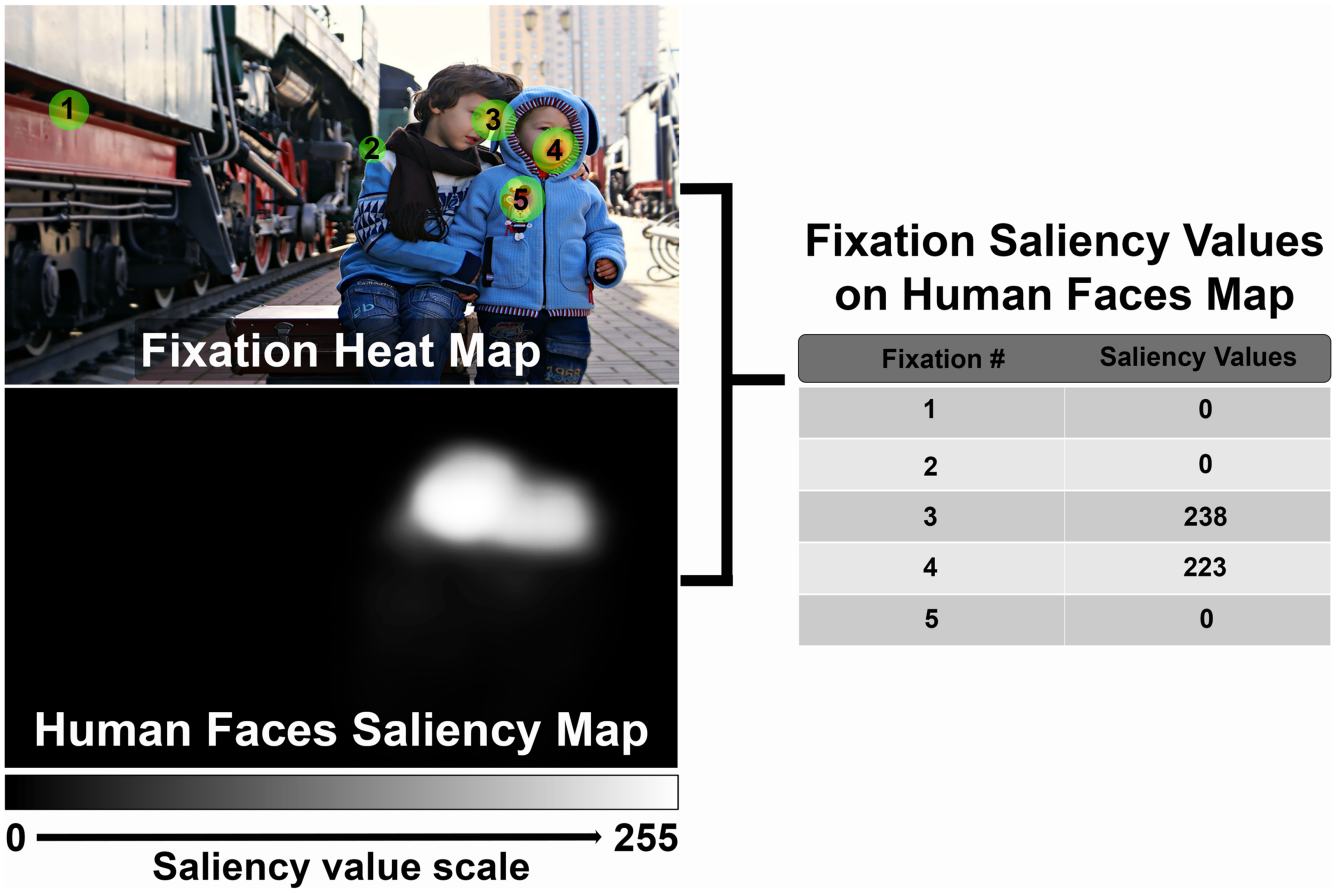
Example demonstrating method to calculate fixation saliency values. See text for full description (Reproduced from Victoria Borodinova via https://www.publicdomainpictures.net/en/index.php, licensed under CC0).

For each image viewed during each eye tracking recording session, fixations will be identified by the eye tracking software as per the above specifications (gaze within 0.1 degree for a minimum of 100 ms). The SR Research Eyelink software uses a heuristic filter to reduce noise (Stampe, 1993). Fixations will be discarded if they are located at least 20% out of the stimulus image range. Trials without valid fixations will be discarded. If both eyes are recorded, the position of the two eyes will be averaged to calculate a composite fixation position. The center of each fixation will be mapped to the corresponding pixel location in the saliency map of interest, and the intensity of this pixel will be designated the fixation saliency value (Figure 3). We will then calculate the average fixation saliency value per image for each participant. Finally, for each saliency characteristic, we will compare fixation saliency values in CVI participants and controls using Mann–Whitney tests. We will also perform multivariate regression to assess the effects of age and neurologic and ophthalmologic comorbidities on fixation saliency values. We will perform subgroup analysis excluding participants with ophthalmologic conditions such as optic atrophy and nystagmus to assess the impact of these factors on our results. We will also perform subgroup analysis by both chronological and developmental age.

#### 2.5.3 Correlation of CVI fixation saliency values to CVI range-CR scores

In children with CVI, we will use Spearman’s correlation coefficient to evaluate the relationship between fixation saliency values and scores of corresponding items on the CVI Range-CR in children with CVI. Specifically, fixation values on the color saliency maps will be correlated to the “color preference” item on the CVI Range-CR. Similarly, fixation values on the visual field saliency maps will be correlated to the “visual field preference” item of the CVI Range-CR. Fixation values on the complexity saliency map will be correlated to scores on the “difficulties with visual complexity” CVI Range-CR item. We will also correlate fixation values on the luminance saliency map with the “need for light” item on the CVI Range-CR. Finally, we will correlate fixation saliency values on the DeepGazeII saliency maps (which indicate visual attention in typical adults) to overall CVI Range-CR scores (Score 1 and Score 2). Correlations will be performed using the whole dataset as well as subgroups divided by age.

## 3 Anticipated results

### 3.1 Saliency maps

We anticipate that by applying adequate prompting engineering, we will be able to generate saliency maps using SegCLIP that accurately represent the visual features that we are investigating. Our preliminary saliency maps are pointing toward this direction (Figure 1). We will continue to refine our prompts to generate appropriate maps for the 12 visual characteristics selected for this study.

### 3.2 Comparison of CVI and control participants

**Figure 4:**
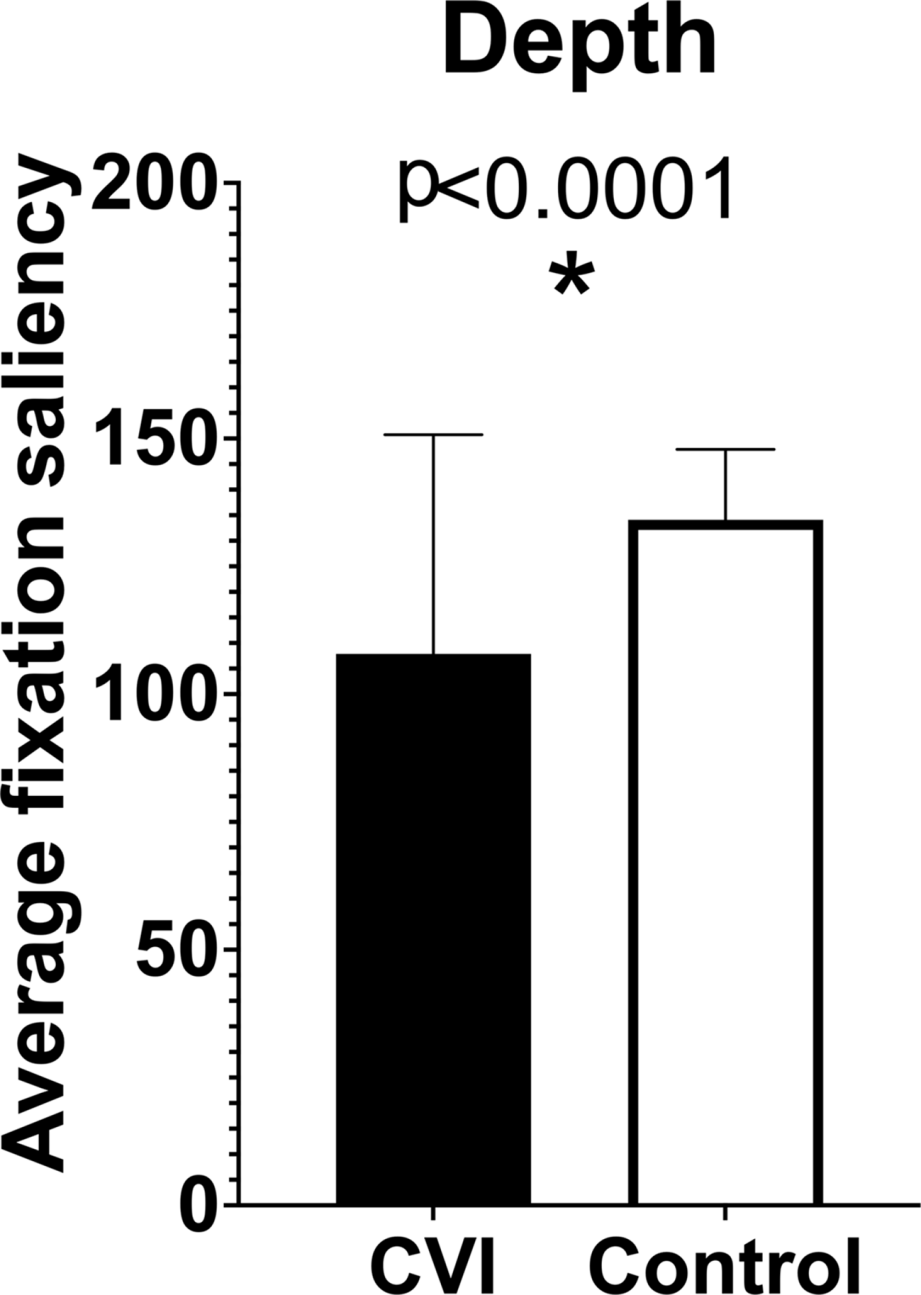
Preliminary results comparing fixation saliency values in children with cerebral/cortical visual impairment (CVI) and age-matched controls on “depth” saliency maps.

We expect that our data will demonstrate significant differences between children with CVI and controls. Specifically, we expect that children with CVI will have lower fixation saliency values for visual characteristics that require higher-order visual processing, including still depictions of motion, two-dimensional representations of depth, human faces and bodies, animals, and visually complex scenes. Our preliminary data suggest that fixation saliency values on the “depth” saliency map are significantly lower in children with CVI compared to controls (Figure 4). We also expect that children with CVI will exhibit greater attention to lower-order visual characteristics, indicating greater reliance on visual features that do not require higher-level processing. Specifically, we anticipate that children with CVI will have higher fixation saliency values on maps of color (especially red and yellow), contrast, orientation, and luminance. Due to eccentric gaze preference and possible visual field defects, children with CVI are also expected to have higher fixation saliency values on peripheral visual field maps. We also anticipate that children with CVI will have difficulty identifying the subject of each image, so we expect that they will have higher fixation saliency values on the background saliency map. On subgroup analysis by age, we hypothesize that older children may have less severe abnormalities in fixation saliency values compared to younger children, based on prior studies reporting improvement in visual behavior over time in children with CVI (Handa et al., 2018).

### 3.3 Correlation of CVI fixation saliency values to CVI range-CR scores

In children with CVI, we anticipate that fixation saliency values will correlate significantly to scores on corresponding items on the CVI Range-CR. Further, we expect that fixation values on the DeepGazeII saliency map will demonstrate significant correlations to the CVI Range-CR overall scores (Score 1 and Score 2).

### 3.4 Advantages

Our saliency modeling approach to evaluate eye fixations has multiple advantages. The approach is quantitative, objective, and scalable, since eye tracking can be performed by technicians or research assistants in approximately 15 min (depending on the child’s cooperation). This is in contrast to functional vision assessments that require trained personnel and may last an hour or more. Because the children are not required to do any task other than watch the computer monitor (free viewing), the method is applicable to young, developmentally delayed children with CVI who are cognitively or physically unable to participate in neuropsychological testing. Additionally, this method takes advantage of generative AI, which can adapt and learn complex or higher-level patterns to generate predictions for subtle features that might otherwise be overlooked (Kümmerer et al., 2016). Therefore, the saliency maps that we generate for this project may improve and become more discriminative of CVI patients compared to controls over time. To ensure that the models remain representative of salient features, we can monitor updates in generative models, re-training and re-evaluating by experts as needed. Additionally, this method is scalable and can incorporate any number of image stimuli for any feature of interest, avoiding costly annotation procedures that would otherwise be done manually.

### 3.5 Limitations

The primary limitation of eye tracking methods in children with CVI is that a minimum level of visual acuity is required to view the stimuli on the computer monitor. We will only include patients with binocular visual acuity of at least 3 cpd, based on Fourier analysis of the images in our experimental protocol (see Participants section above). Thus, our study may not be applicable to children with worse visual acuity (i.e., grating acuity lower than 3 cpd). Since we will exclude participants with oculomotor apraxia, our results may also be inapplicable to children with this condition. Oculomotor apraxia has been reported in 15% of children with CVI (Huo et al., 1999). Additionally, the visual stimulus and testing conditions involving two-dimensional images on a computer monitor in a laboratory may not fully simulate real-life situations. Furthermore, we will use the CVI Range-CR to validate our eye tracking findings, but the validity of CVI Range-CR is still under investigation (Chang et al., 2022). We chose to compare eye tracking to CVI Range-CR scores since there are no other direct assessments for children with CVI that quantify the lower- and higher-order visual characteristics evaluated in this study and are applicable to our population of young, developmentally delayed patients. When there is no gold standard reference test, comparison to other relevant clinical tests or characteristics is an acceptable alternative for preliminary validation (Rutjes et al., 2007).

Another limitation relates to the use of generative AI to create the saliency maps. These AI models can “hallucinate” and generate outputs that may be inaccurate. Therefore, the generated maps require human review to confirm that they are consistent with the visual characteristics that are prompted. Careful attention to prompt engineering and some degree of trial-and-error may be required to generate accurate saliency maps for all characteristics.

Furthermore, we will screen typically developing control participants for neurologic and neurodevelopmental conditions based on birth and medical history. We cannot exclude the possibility that some of these children may have undiagnosed neurodevelopmental conditions such as autism or attention deficit hyperactivity disorder (ADHD). However, they will have a screening eye exam to confirm normal visual function.

Finally, patterns of eye tracking are indicative of visual attention, and we will infer deficits of visual processing when there is decreased visual attention to features that require specific levels of visual processing. However, since we anticipate that all of our CVI participants will be non-verbal or minimally verbal, we will be unable to confirm whether decreased attention is due to abnormalities of visual perception or other factors. For example, children with autism exhibit decreased visual attention to social stimuli on eye tracking (Chita-Tegmark, 2016), which is believed to be related to social rather than visual deficits. Future eye tracking studies in individuals with CVI with greater communication abilities may help to clarify the interpretation of our results.

### 3.6 Potential pitfalls and alternate approaches

Eye tracking in patients with strabismus requires some modifications. A trained examiner must determine the fixating eye and further assess whether fixation alternates, in order to determine which eye to record from and whether monocular occlusion is necessary (in the case of alternating fixation). Monocular occlusion may not be possible in some children with CVI. If monocular occlusion is needed, we will first attempt this using an adhesive eye patch. If the child wears glasses, occlusion of one lens is an option. If they cannot tolerate this, we will request that that parent cover one eye with a hand, being careful not to allow peeking. If these measures fail, then the child will be excluded from our study.

Furthermore, nystagmus may interfere with accurate detection of gaze direction. Nystagmus in CVI patients generally occurs only in the presence of anterior visual pathway disease (Whiting et al., 1985). Since the only intraocular comorbidity that we are allowing for this study is optic atrophy, we anticipate that only a minority of participants will have nystagmus. Our experience is that if nystagmus amplitude and frequency are low enough to enable calibration, then we can accurately assess the location of fixations during the eye tracking recording (the timing of fixations and saccades, however, will not be reliable). Therefore, we will include patients with nystagmus who are able to complete calibration in this study. We will perform subgroup analysis excluding participants with nystagmus to determine the impact of nystagmus on saliency results.

## 4 Discussion

Eye tracking with interpretation enabled by AI-generated saliency maps has the potential to serve as a quantitative and objective metric of attention to lower- and higher-order visual characteristics in children with CVI. Fixation saliency values may potentially be used in the future for longitudinal assessments and guidance of personalized interventions. For example, if a color preference is identified based on high fixation saliency values on a certain color saliency map, then the family may be suggested to use objects of this color to encourage visual behavior. Additionally, because this technique is scalable due to minimal time and personnel requirements compared to other methods of visual assessment in CVI, eye tracking is an ideal candidate to serve as an outcome measure in future multi-center clinical trials to identify evidence-based medical treatments. Finally, successful application of eye tracking combined with generative AI for visual assessment in CVI could lead to adoption of this technique in other neurodevelopmental disorders.

## Data Availability

The original contributions presented in the study are included in the article/Supplementary material, further inquiries can be directed to the corresponding author.
