# Supplementary material for: Method for assessing visual saliency in children with cerebral/cortical visual impairment using generative artificial intelligence
Source: Front Hum Neurosci. 2025 Jan 17;18:1506286. doi: 10.3389/fnhum.2024.1506286 (PMC11782282; doi:10.3389/fnhum.2024.1506286)
Supplement: Supplementary file 1 [file Table_1.DOCX]

# Supplemental Data. CVI Range-CR Scoring: Across-CVI and Within-CVI Characteristics Assessment Methods. Reproduced from BMJ Open Ophthalmology, Chang M, Roman-Lantzy C, O'Neil SH, et al., 7, 1-8, 2022, with permission from BMJ Publishing Group Ltd.

# The CVI Range: Across-CVI Characteristics Assessment Method

**CVI Range 1-2: Student functions with minimal visual responses**

| **O** | **I** | **D** | R | + | +/- | - |  |
| --- | --- | --- | --- | --- | --- | --- | --- |
|  |  |  |  |  |  |  | May localize, but no appropriate fixations on  objects or faces |
|  |  |  |  |  |  |  | Consistently attentive to lights or perhaps ceiling  fans |
|  |  |  |  |  |  |  | Prolonged periods of latency in visual tasks |
|  |  |  |  |  |  |  | Responds only in strictly controlled environments |
|  |  |  |  |  |  |  | Objects viewed are a single color |
|  |  |  |  |  |  |  | Objects viewed have movement and/or shiny or  reflective properties |
|  |  |  |  |  |  |  | Visually attends in near space only |
|  |  |  |  |  |  |  | No blink in response to touch or visual threat |
|  |  |  |  |  |  |  | No regard of the human face |

**CVI Range 3-4: Student functions with more consistent visual response**

| **O** | **I** | **D** | R | + | +/- | - |  |
| --- | --- | --- | --- | --- | --- | --- | --- |
|  |  |  |  |  |  |  | Visually fixates when the environment is  controlled |
|  |  |  |  |  |  |  | Less attracted to lights: can be redirected |
|  |  |  |  |  |  |  | Latency slightly decreases after periods of  consistent viewing |
|  |  |  |  |  |  |  | May look at novel objects if they share  characteristics of familiar objects |
|  |  |  |  |  |  |  | Blinks in response to touch and/or visual threat, but the responses may be latent and/or  inconsistent |
|  |  |  |  |  |  |  | Has “favorite” color |
|  |  |  |  |  |  |  | Shows strong visual field preferences |
|  |  |  |  |  |  |  | May notice moving objects at 2 to 3 feet |
|  |  |  |  |  |  |  | Look and touch completed as separate events |

**CVI Range 5-6: Student uses vision for functional tasks**

| **O** | **I** | **D** | R | + | +/- | - |  |
| --- | --- | --- | --- | --- | --- | --- | --- |
|  |  |  |  |  |  |  | Objects viewed may have two to three colors |
|  |  |  |  |  |  |  | Light is no longer a distractor |
|  |  |  |  |  |  |  | Latency present only when the student is tired,  stressed, or overstimulated |
|  |  |  |  |  |  |  | Movement continues to be an important factor for  visual attention |
|  |  |  |  |  |  |  | Student tolerates low levels of background noise |
|  |  |  |  |  |  |  | Blink response to touch is consistently present |
|  |  |  |  |  |  |  | Blink response to visual threat is intermittently  present |
|  |  |  |  |  |  |  | Visual attention now extends beyond near space,  up to 4 to 6 feet |
|  |  |  |  |  |  |  | May regard familiar faces when voices do not compete |

**CVI Range 7-8: Student demonstrates visual curiosity**

| **O** | **I** | **D** | R | + | +/- | - |  |
| --- | --- | --- | --- | --- | --- | --- | --- |
|  |  |  |  |  |  |  | Selection of toys or objects is less restricted;  requires one to two sessions of “warm up” |
|  |  |  |  |  |  |  | Competing auditory stimuli tolerated during periods of viewing; the student may now maintain  visual attention on objects that produce music |
|  |  |  |  |  |  |  | Blink response to visual threat consistently  present |
|  |  |  |  |  |  |  | Latency rarely present |
|  |  |  |  |  |  |  | Visual attention extends to 10 feet with targets  that produce movement |
|  |  |  |  |  |  |  | Movement not required for attention at near  distance |
|  |  |  |  |  |  |  | Smiles at/regards familiar and new faces |
|  |  |  |  |  |  |  | May enjoy regarding self in mirror |
|  |  |  |  |  |  |  | Most high-contrast colors and/or familiar patterns  regarded and interpreted |
|  |  |  |  |  |  |  | Simple books, picture cards, or symbols regarded and interpreted |

**CVI Range 9-10: Student spontaneously uses vision for most functional activities at level approaching near typical**

| **O** | **I** | **D** | R | + | +/- | - |  |
| --- | --- | --- | --- | --- | --- | --- | --- |
|  |  |  |  |  |  |  | Selection of toys or objects not restricted to the  familiar; visually curious in new settings |
|  |  |  |  |  |  |  | Only the most complex environments affect  visual response |
|  |  |  |  |  |  |  | Latency never present |
|  |  |  |  |  |  |  | No color or pattern preference |
|  |  |  |  |  |  |  | Visual attention and interpretation extends  beyond 20 feet |
|  |  |  |  |  |  |  | Views and interprets information from non-backlit two-dimensional materials and simple images |
|  |  |  |  |  |  |  | Uses vision to imitate actions |
|  |  |  |  |  |  |  | Demonstrates memory of visual events |
|  |  |  |  |  |  |  | Displays typical visual-social responses |
|  |  |  |  |  |  |  | Visual fields unrestricted |
|  |  |  |  |  |  |  | Look and reach completed as a single action |
|  |  |  |  |  |  |  | Views and interprets information from non-backlit two-dimensional images presented on complex, visually dense backgrounds |

**The CVI Range: Within-CVI Characteristics Assessment Method**

Determine the level of CVI present or resolved in the 10 categories below and add to obtain total score. Rate the following CVI categories as related to the student/child’s visual behaviors by circling the appropriate number (the CVI Progress Chart may be useful as a scoring guide):

**0** Full effect of the characteristic is present

**.25** Behavior on this characteristic has begun to change or improve

**.5** The characteristic is affecting visual functioning approximately half the time

**.75** Occasional effect of the characteristic; response is nearly like that of individuals the same age

**1** Resolving, approaching typical, or response is the same as others of the same age

| **1. Color Preference** | **0** | **.25** | **.5** | **.75** | **1** |
| --- | --- | --- | --- | --- | --- |
| **2. Need for movement** | **0** | **.25** | **.5** | **.75** | **1** |
| **3. Visual latency** | **0** | **.25** | **.5** | **.75** | **1** |
| **4. Visual field preferences** | **0** | **.25** | **.5** | **.75** | **1** |
| **5. Difficulties with visual complexity- object**  **array**  **sensory**  **faces** | **0**  **0**  **0**  **0**  **0** | **.25**  **.25**  **.25**  **.25**  **.25** | **.5**  **.5**  **.5**  **.5**  **.5** | **.75**  **.75**  **.75**  **.75**  **.75** | **1**  **1**  **1**  **1**  **1** |
| **6. Need for light** | **0** | **.25** | **.5** | **.75** | **1** |
| **7. Difficulty with distance viewing** | **0** | **.25** | **.5** | **.75** | **1** |
| **8. Atypical visual reflexes** | **0** | **.25** | **.5** | **.75** | **1** |
| **9. Difficulty with visual novelty** | **0** | **.25** | **.5** | **.75** | **1** |
| **10. Absence of visually guided reach** | **0** | **.25** | **.5** | **.75** | **1** |
